# A Novel Low-Cost Fibrous Tempered-Martensite/Ferrite Low-Alloy Dual-Phase Steel Exhibiting Balanced High Strength and Ductility

**DOI:** 10.3390/ma18061292

**Published:** 2025-03-14

**Authors:** Xianguang Zhang, Yiwu Pei, Haoran Han, Shouli Feng, Yongjie Zhang

**Affiliations:** 1 School of Metallurgical and Ecological Engineering, University of Science and Technology Beijing, Beijing 100083, China; 2 Institute for Materials Research, Tohoku University, 2-1-1 Katahira, Aoba-ku, Sendai 980-8577, Japan

**Keywords:** dual-phase steel, medium-carbon steel, austenite reversion, intercritical annealing, tempered martensite, fine carbide precipitation, high-density dislocations, nanotwins, strength–ductility balance

## Abstract

Low-cost and low-alloy dual-phase (DP) steel with a tensile strength (TS) above 1000 MPa and high ductility is in great demand in the automobile industry. An approach to using a medium-carbon and fibrous DP structure for developing such new DP steel has been proposed. The microstructure and mechanical performance of fibrous DP steel obtained via partial reversion from martensite in Fe-C-Mn-Si low-alloy steel have been investigated. The TS of the as-quenched DP steel is above 1300 MPa, while the total elongation is less than 6%. The total elongation was increased to above 13%, with an acceptable loss in TS by performing additional tempering. The fibrous tempered-martensite/ferrite DP steel exhibits an excellent balance of strength and ductility, surpassing the current low-alloy DP steels with the same strength grade. Plate-like or quasi-spherical fine carbides were precipitated, and the relatively high-density dislocations were maintained due to the delay of lath recovery by the enrichment of Mn and C in martensite (austenite before quenching), contributing to the tempering softening resistance. In addition, nanotwins and a very small amount of retained austenite were present due to the martensite chemistry. High-density dislocations, fine carbide precipitation, and partially twinned structures strengthened the tempered martensite while maintaining relatively high ductility. Quantitative strengthening models and calculations were not included in the present work, which is an interesting topic and will be studied in the future.

## 1. Introduction

Dual-phase (DP) steel is widely used in car body structural parts such as automobile brackets, brake disks, cross beams, A/B/C pillars, and bumper reinforcements [1,2,3,4,5]. Improving the strength and ductility of DP steel is essential for automobile lightweighting and reduction in CO_2_ emissions. High-strength DP steel with tensile strength above 1000 MPa and high ductility is in great demand in the automotive industry.

Various grades of high-strength DP steels have been developed, such as DP 1180 [6,7,8] and DP 1300 [9,10]. Micro-alloying elements, such as V, Nb, or Ti, or complex alloying additions, such as Cr, Mo, and Cu, are usually added to low-alloy DP steels to increase their strength [11,12,13,14,15,16], which significantly increases the cost. On the other hand, the ductility is mostly lower than 10% [7,8,9,10], which limits their applications. In addition to increasing strength, improving ductility is crucial for ensuring excellent formability and good absorption of collision energy.

In recent years, many efforts have been made to produce DP steels with a balance of high strength and ductility. For example, austenite/martensite DP steel with ductility of ~30% and tensile strength over 1400 MPa was developed by designing an alloy with the main composition of Fe-9Mn-4Ni-1Al-2.5Cu-1.5Mo-1.5W (mass%) [17]. Additionally, medium Mn steels with 5–8% Mn (mass%) were developed, achieving tensile strengths above 1000 MPa and total elongation above 38% [18,19,20]. However, the content of alloying elements remains high, resulting in higher costs compared to low-alloy DP steels. Furthermore, producing high-alloy steel in the industry still presents some challenges. Therefore, it is crucial to develop low-cost, high-strength, and high-ductility low-alloy DP steels.

Traditional DP steels are produced through cold-rolling and hot-rolling processes, resulting in structures composed of equiaxed martensite and ferrite. Recently, it has been reported that fibrous low-alloy and low-carbon DP steel, with an alternate layer distribution of lamellar martensite/ferrite structures obtained via partial austenite reversion from martensite, exhibits an excellent balance of strength and ductility, as well as cracking resistance [21,22,23]. This is attributed to the regular and uniform martensite/ferrite structures. Although the tensile strength of fibrous DP steel is lower than 1050 MPa, it provides a new opportunity to produce high-strength and high-ductility low-alloy DP steels by utilizing the concept of fibrous martensite/ferrite structures.

Moreover, the idea of using universal materials for vehicles has been proposed in recent years to solve the problem of joining between different materials [24]. Fe-C-Mn-Si alloys (usually around 1.5Si mass% was added for suppressing the carbide precipitation during partitioning or austempering treatment) were used for the quenching and partitioning (QP), and transformation induced plasticity (TRIP) aided series advanced high strength steels (AHSS) [25,26,27,28,29], which are the important materials for the car body as well. However, the high-strength fibrous DP steel with the composition of QP and TRIP-aided series AHSS has not yet been well studied, and the mechanical performance of medium-carbon fibrous DP steel is unclear. Therefore, the present work aims to clarify the possibility of developing a Fe-C-Mn-Si low-alloy fibrous DP steel without micro-alloying element additions, with consideration of low cost, high strength and ductility, and a unified material for joining. In this work, the mechanical performance of a low-cost Fe-C-Mn-Si fibrous DP steel without expensive micro-alloying element additions was studied.

## 2. Materials and Methods

### 2.1. Material Preparation

**Figure 1:**
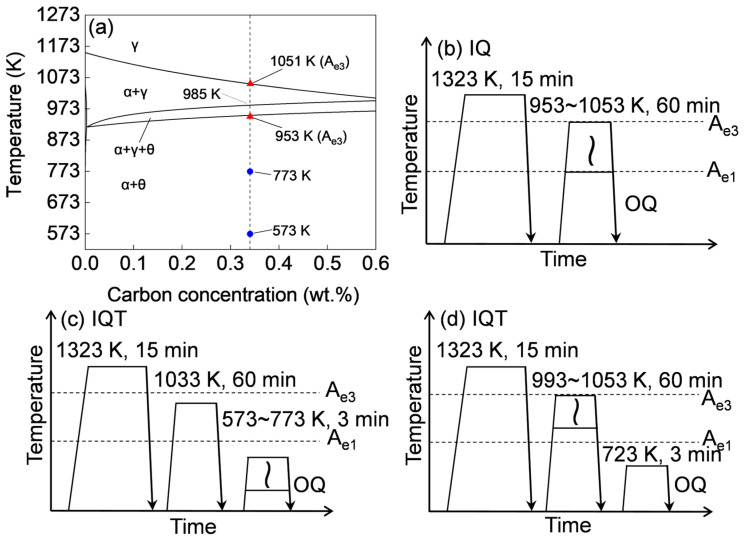
(**a**) Equilibrium phase diagram of the Fe-2.51Mn-1.47Si-C alloy system; schematic illustrations of the heat-treatments for IQ-DP steels (**b**) under varying intercritical annealing temperatures and IQT-DP steels under varying (**c**) tempering temperatures and (**d**) intercritical annealing temperatures. The blue points indicate the tempering temperature range.

A Fe-0.34C-1.47Si-2.51Mn low alloy medium carbon steel with a similar composition to the QP or TRIP steels was used in this work. The detailed chemical composition of the steel is given in Table 1. The equilibrium phase diagram calculated by ThermoCalc TCFE9 software is shown in Figure 1. The A_e1_ and A_e3_ temperatures were calculated to be 953 K and 1051 K, respectively, as indicated in the phase diagram in Figure 1a. The alloy was hot rolled into a 20 mm thick plate after being smelted in a medium frequency vacuum induction melting furnace and then cold rolled to a 1.5 mm thin sheet.

### 2.2. Heat Treatment

The thermal history for the heat treatment is shown in Figure 1b–d. The thin plate specimens with a size of 1.5 × 40 × 120 mm taken along the rolling direction of the cold-rolled steel sheet were used for the heat treatment. The cold-rolled thin-plate specimens were austenitized at 1323 K for 15 min in a vacuum tube furnace, and then oil quenched (the average cooling rate is approximately 300 K·s^−1^) to room temperature to obtain as-quenched martensite. Here, moderate oil-quenching was used instead of water-quenching to avoid bending the thin-plate sample due to the introduction of high internal stress. Then, some of the as-quenched martensite samples were intercritically annealed at varying temperatures ranging from 953 K to 1053 K for 60 min and oil-quenched to room temperature to produce DP steels, which was referred to as IQ-DP (Figure 1b). After that, some of the IQ-DP steels were tempered at varying temperatures ranging from 573 K to 773 K for 180 s (Figure 1c) or intercritically annealed at varying temperatures ranging from 953 K to 1053 K and tempered at a fixed condition, which were referred as IQT-DP steels (Figure 1d).

### 2.3. Mechanical Testing

The tensile specimens were cut from the heat-treated samples and prepared according to ASTM E8/E8M-24 standard [31] with a gauge length of 25 mm and a width of 6 mm. The tensile tests were performed at room temperature using a universal testing machine at a crosshead speed of 1 mm·min^−1^ monitored by the extensometer. The tensile tests were repeated three times to ensure reproducibility. Vickers’ hardness tester was used for hardness tests according to ASTM E384-22 standard [32].

### 2.4. Microstructural Characterization

The samples were mechanically polished with different grades of sandpaper and polishing cloth, and then the polished samples were treated with 3% nitric alcohol (Sinopharm Chemical Reagent Co., Ltd., Shanghai, China) for 5~10 s. The microstructure was characterized by field emission–scanning electron microscope (FE-SEM; JEOL, JSM-6071F, Tokyo, Japan) and scanning transmission electron microscopy (STEM, Titan ETEM G2, Hillsboro, OR, USA) equipped with EDS operated at 300 kV. Twenty SEM images with a magnification of 5000 times were taken for each heat treatment sample of the IQ series, and the volume fraction of austenite (transformed into martensite after quenching) was counted quantitatively by the metallographic point counting method.

## 3. Results

### 3.1. Microstructure of IQ-DP Steels

**Figure 2:**
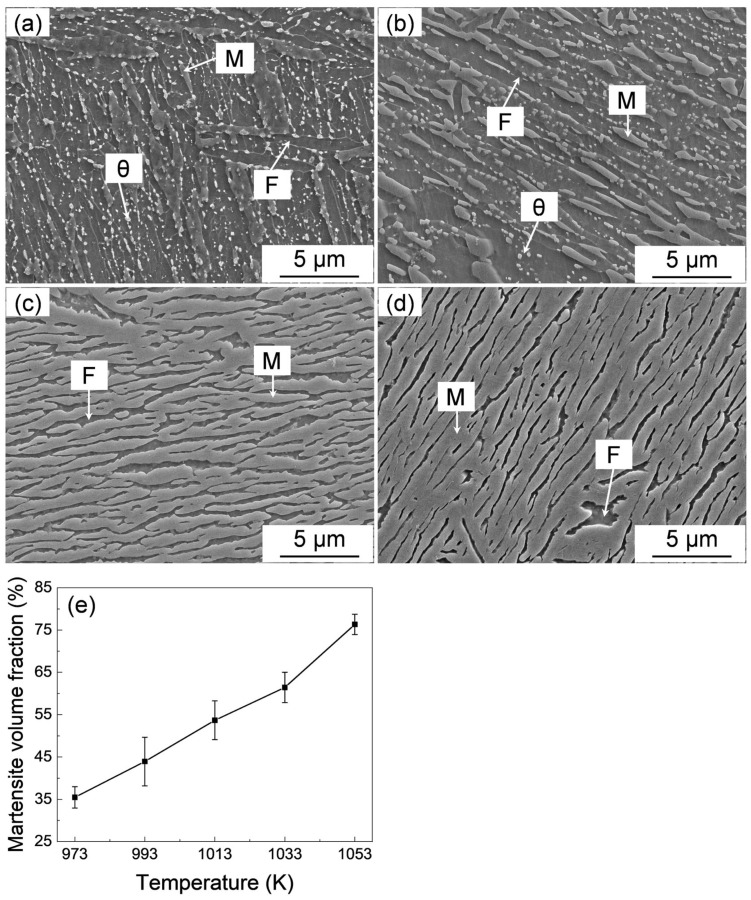
(**a**–**d**) SEM images of IQ-DP steels after intercritical annealing at (**a**) 953 K, (**b**) 973 K, (**c**) 993 K, (**d**) 1033 K, and (**e**) the quantified martensite volume fraction of IQ-DP steels after intercritical annealing at varying temperatures. F, M, and θ represent ferrite, martensite, and cementite, respectively.

The microstructure evolution during intercritical annealing (IA) at various temperatures is shown in Figure 2a–d. A typical tempered martensite structure, which is composed of cementite particles and lath tempered-martensite (ferrite) matrix, is observed after annealing at 953 K for 60 min (Figure 2a). Acicular-shaped fresh martensite (austenite before quenching), which is distributed in a tempered martensite matrix with undissolved cementite particles, is observed after annealing at 973 K for 1 h (Figure 2b). The amount of acicular austenite gradually increases and thickens, accompanying the dissolution of cementite particles with the increase in annealing temperature. The volume fraction of fresh martensite quantitatively analyzed by metallographic point-counting method [33] is summarized in Figure 2e. With the increase in intercritical annealing temperature, the volume fraction of martensite gradually increased from about 35% to 75%.

### 3.2. Mechanical Properties of IQ-DP Steels

**Figure 3:**
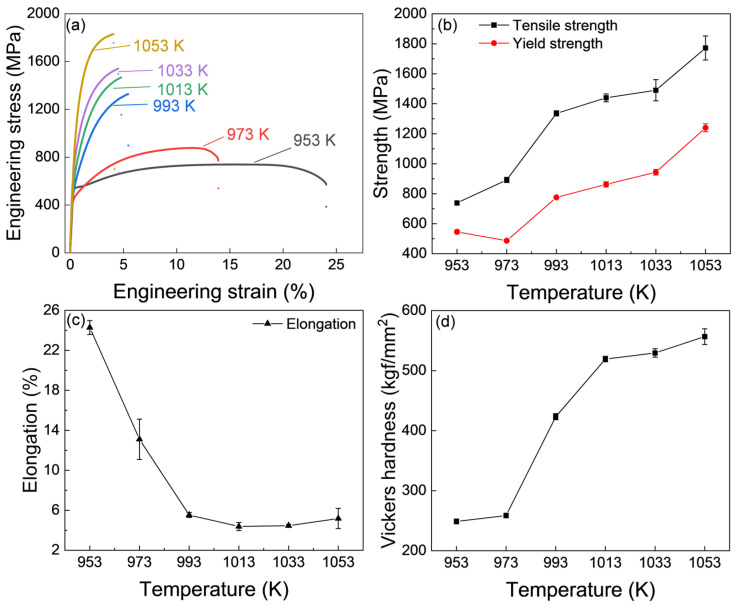
(**a**–**c**) Mechanical properties and (**d**) hardness of IQ-DP steels after intercritical annealing at varying temperatures.

The tensile curves of the IQ-DP steels after intercritical annealing at varying temperatures are shown in Figure 3a, and the changes in yield tensile strength and total elongation against the intercritical annealing temperature are summarized in Figure 3b,c. The IQ-DP steels, after annealing at 953 K, exhibited a relatively low strength with high ductility. This is due to the negligible fresh martensite formed and the formation of soft and ductile tempered martensite (ferrite), as shown in Figure 2a. As the annealing temperature was increased to 973 K, the strength slightly increased while the ductility decreased, which is attributed to the formation of 35% fresh martensite (Figure 2b,e). With the further increase in annealing temperature up to 1053 K, both the yield and tensile strength gradually increased and reached as high as 1800 MPa, while the elongation dramatically decreased to lower than 5%. The Vickers hardness of the series IQ-DP steels is plotted in Figure 3d. With the increase in intercritical annealing temperature, the hardness gradually increased, which exhibited a similar changing tendency to that in tensile strength and martensite volume fraction.

The intercritical annealing at 973 K with around 35% martensite does not have an obvious advantage in mechanical properties compared with traditional similar-grade DP steels [34,35]. There is a sudden change in strength and ductility as the IA temperature is increased from 973 K to 993 K. Therefore, it is difficult to produce high strength and ductility balanced DP steel for the medium carbon steel with fibrous martensite + ferrite structures, which is different from that of low-carbon fibrous-DP steel exhibiting much higher strength and ductility balance than that of the traditional equiaxed DP steels [22].

### 3.3. Mechanical Properties of IQT-DP Steels

According to the above results, although the tensile strength of IQ-DP steel is above 1300 MPa, the total elongation is less than 6%, which is a restriction for the application. Hence, additional tempering is necessary to increase ductility. The IQ-DP steel annealed at 1033 K was selected as an example for studying the influences of tempering temperature ranging from 573 K to 773 K on the mechanical properties and microstructures of IQT-DP steels.

**Figure 4:**
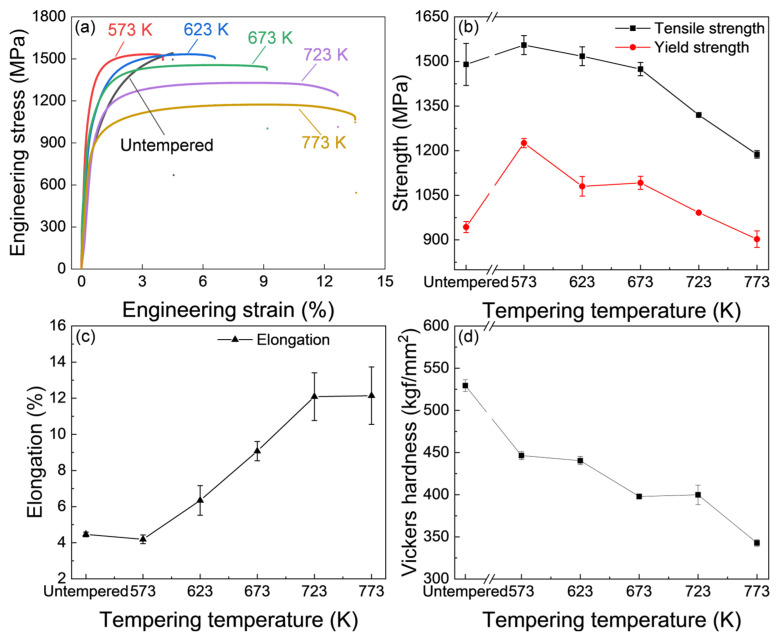
(**a**–**c**) Mechanical properties and (**d**) hardness of IQT-DP steels after intercritical annealing at 1033 K and tempering at varying temperatures.

The tensile properties of the IQT-DP steels after tempering at various temperatures are shown in Figure 4a, in which the IQ-DP steel (untempered case) was plotted as a reference. The detailed mechanical properties are summarized in Figure 4b,c. The ultimate tensile strength (UTS) of IQT-DP steels is gradually decreased from 1555.1 MPa to 1187.7 MPa (Figure 4b), while the total elongation is apparently increased from 4.2% to 13.6% (Figure 4c), as the tempering temperature is raised from 573 K to 773 K. For example, the total elongation of the tempered IQ-T series has been improved from 4.6% of IQ-DP to 13.6% of IQT-DP. In addition, the yield strength is firstly increased and then decreased with the increase in tempering temperature, while the yield strength of the IQT-DP steels is even higher than that of the IQ-DP case as the tempering temperature is increased up to 723 K, which should be related to the variation in microstructures. The corresponding changes in Vickers-hardness are shown in Figure 4d. The hardness gradually decreases, which shares the same changing tendency as that of UTS against tempering temperature due to the softening effect. According to the above results, tempering at 723 K produced a relatively excellent strength and ductility balance. Therefore, the tempering at 723 K was selected for studying the influences of intercritical annealing temperatures on the mechanical responses of DP steel after tempering.

### 3.4. Strength–Ductility Comparison

**Figure 5:**
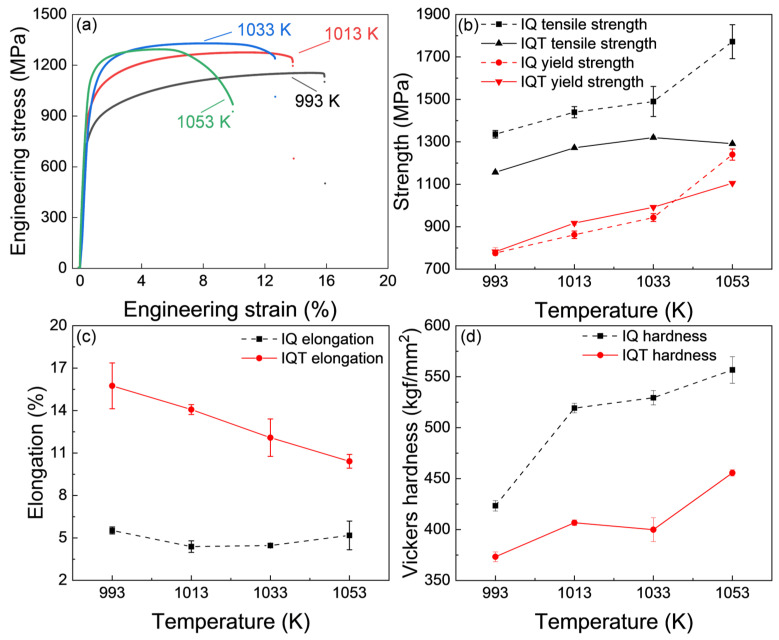
(**a**–**c**) Mechanical properties and (**d**) hardness of IQT-DP steels after intercritical annealing at different temperatures and tempering at 723 K.

The influences of intercritical-annealing temperatures on the tensile properties of the IQT-DP steels are shown in Figure 5a–c, and the changes in strength and elongation against annealing temperature for the untempered (IQ-DP) case were plotted as well for comparison. Both the tensile and yield strength of the IQT-DP steels increases with the increase in intercritical annealing temperature while the total elongation gradually decreases. In addition, the intercritical annealing at 1053 K shows the lowest total elongation, suggesting that over 70% fresh martensite is superfluous for the DP steel to obtain an excellent balance of mechanical properties.

In addition, it can be noticed that the yield strength of the IQT-DP steels is higher than that of the IQ-DP steels at the same IA temperature above 1013 K, and the total elongation is greatly improved. Although there is a reduction in UTS, it is still above 1200 MPa. The changes in Vickers hardness against the change in annealing time for the I-QT series are shown in Figure 5d. The hardness gradually increases with the increase in intercritical annealing temperature. The Vickers hardness of the IQT series is lower than that of the IQ series at the same IA temperature due to the tempering softening.

**Figure 6:**
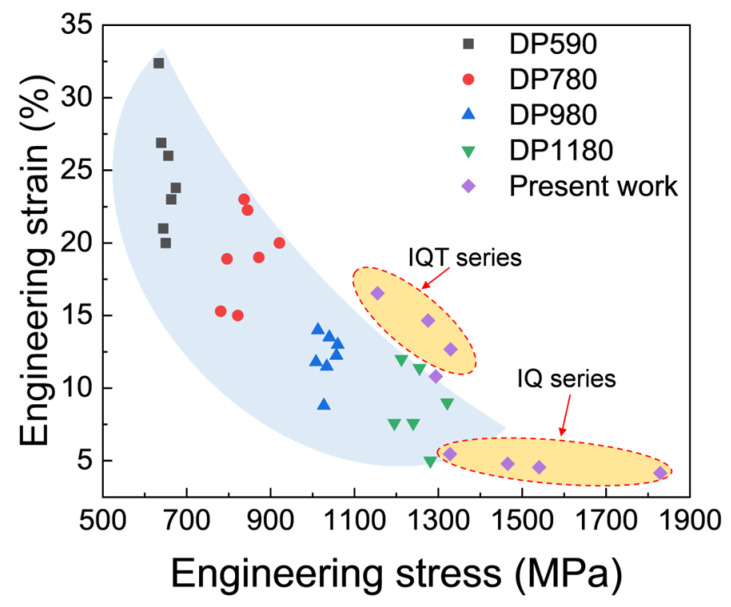
Comparisons in the strength–ductility balance of the IQ series and IQT series DP steels and the reported or commercialized DP steels [8,36,37,38,39,40,41,42].

The strength and ductility balance for the IQ and IQT series of DP steels are displayed in Figure 6, in comparison with those of the commercialized or the literature-reported DP steels [8,36,37,38,39,40,41,42]. The IQT series exhibits a higher ductility than commercialized or industrialized ones, with strength ranging from 1150 MPa to 1300 MPa, and the Fe-C-Mn-Si is a simple and low-cost alloy system. Especially at the strength level of 1200–1300 MPa, the total elongation reached as high as 13%.

## 4. Discussion

According to the above results, a Fe-C-Mn-Si low alloy low-cost fibrous tempered-martensite/ferrite DP steel with excellent strength and ductility beyond the current commercialized and the literature reported low-alloy DP steels was developed without adding expensive alloying elements such as V, Cr or Mo. To understand the mechanisms, a deeper study on microstructures was performed, and the following will be discussed.

**Figure 7:**
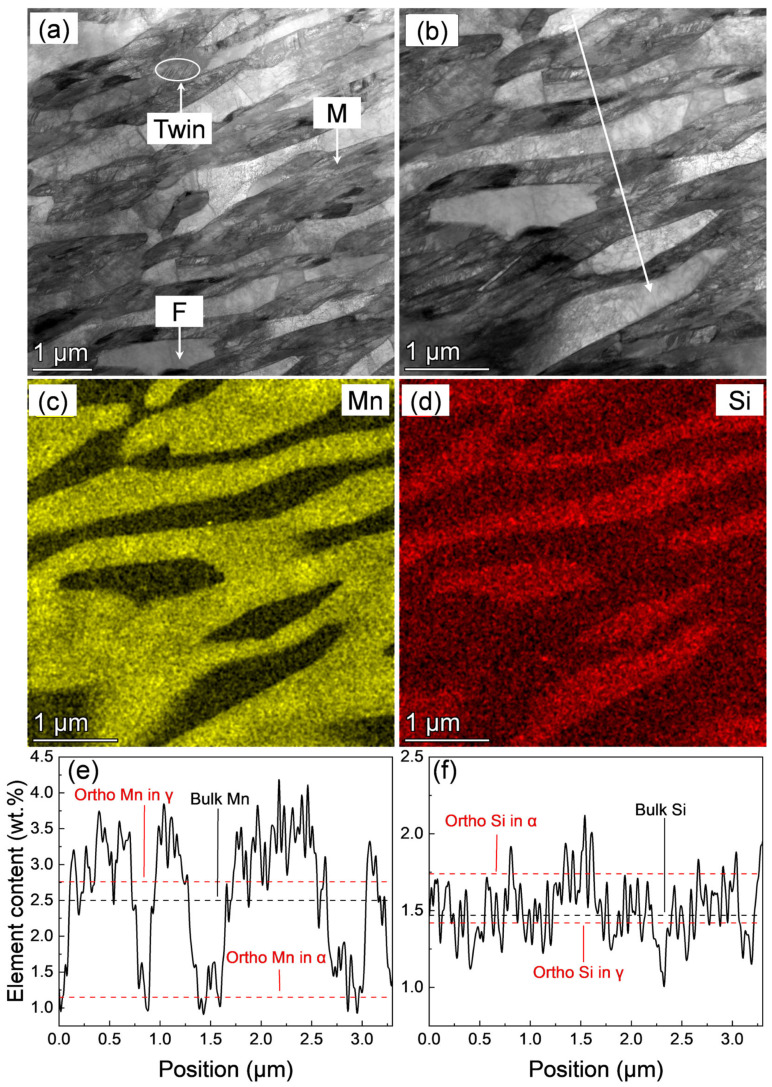
(**a**,**b**) Bright-field images, (**c**,**d**) corresponding Mn and Si STEM-EDS mapping, and (**e**,**f**) quantitative line analyzed results of Mn and Si distribution along the arrows in (**b**) of IQ-DP steel after intercritical annealing at 1033 K for 3600 s.

The bright-field TEM images for the IQ-DP steels after intercritical annealing at 1033 K with around 55% fresh martensite are shown in Figure 7a. Acicular-shaped fresh martensite and ferrite lamellar structures were clearly observed. Lath martensite was dominantly formed, and some of the martensites are internally twinned. This should be related to the Mn and C enrichment during the austenite reversion of intercritical annealing. The bright-field TEM image and corresponding STEM-EDX mapping and line analysis results are shown in Figure 7b–f, in which the equilibrium Mn and Si contents at the temperature of 1033 K were plotted as well. Mn was seriously enriched into the reverted austenite, while Si was slightly depleted. The experimentally measured average Mn and Si contents of the reverted austenite and the equilibrium C content were summarized in Table 2 (the C content was difficult to quantify by STEM-EDS; therefore, the equilibrium C content was used). The higher carbon and Mn contents lead to the twinned substructure of martensite, the same as the twinned martensite formed in the high C and Mn steels [43,44].

**Figure 8:**
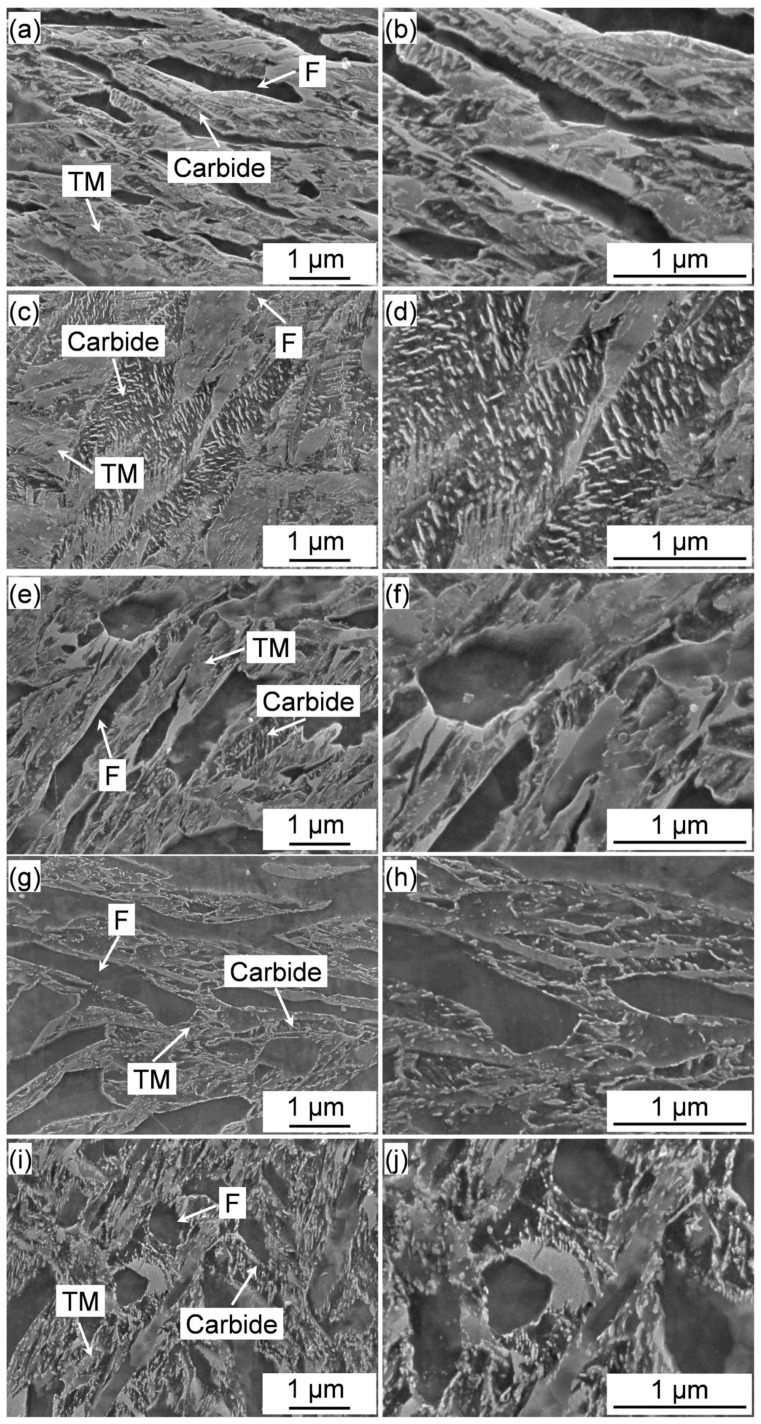
SEM images of the IQT-DP steels after intercritical annealing at 1033 K for 3600 s and tempering at varying temperatures: (**a**,**b**) 573 K; (**c**,**d**) 623 K, (**e**,**f**) 673 K, (**g**,**h**) 723 K, (**i**,**j**) 773 K.

The SEM images of the IQT-DP steels after being tempered at various temperatures are shown in Figure 8. The fresh martensite was decomposed after tempering at 573 K, and the tempered structure is composed of tempered martensite (TM) and carbide. As the tempering temperature increases, the degree of martensite decomposition gradually increases. Plate-like carbides were clearly formed after tempering at 623 K and 673 K. And the carbides tend to be plate-like to quasi-plate-like and sphere with the tempering temperature increased to 723 K and 773 K. It is known that the precipitation of cementite is controlled by diffusion of carbon atoms to lattice defects in the order of PAGB, block boundaries (larger cementite), lath boundaries (fine inter-lath cementite), and dislocations (finer intra-lath cementite) [45]. This is consistent with the location dependence of carbide precipitation and coarsening behavior during the tempering of IQ-DP steel. In addition, the high Mn content suppressed the growth and coarsening of cementite [46]. This caused the relatively slower growth and refined carbide precipitates.

**Figure 9:**
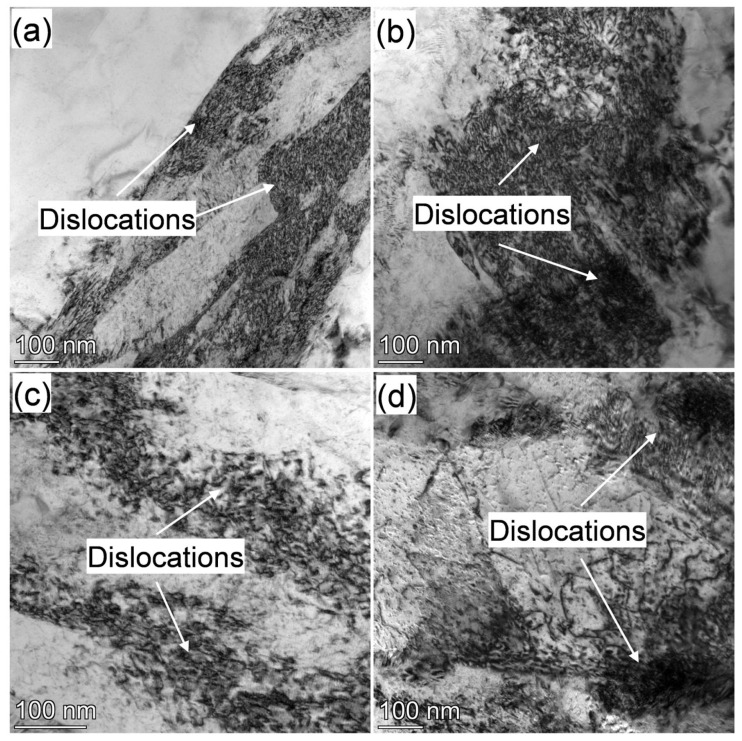
BF TEM images of (**a**) IQ-DP steel after intercritical annealing at 1033 K and IQT-DP steels after tempering at (**b**) 623 K, (**c**) 673 K, and (**d**) 723 K.

TEM analyses were carried out to understand the tempering behavior of martensite deeply. The bright-field TEM images taken at two tilt angles to reveal the dislocations are shown in Figure 9. The white area in the TEM images is the ferrite matrix, and the black part is the area where dislocations (dislocation lines, dislocation cells) are gathered and mainly distributed in martensite. High-density dislocations are observed in the as-quenched fresh martensite (Figure 9a). The high-density dislocations were maintained even after tempering at 623 K for 3 min (Figure 9b). At the same time, the density of dislocations was apparently reduced after tempering above 673 K (Figure 9c,d). This strongly indicates that martensite laths become partially recovered, i.e., a reduction in dislocation density, during the tempering of IQT-DP steel. This also explained the decreases in tensile strength.

The martensite recovery kinetics during the tempering process is generally determined by two key factors: retardation of martensite lath-boundaries migration by the carbides pinning effect [47] and development of martensite laths recovery against time, i.e., dislocations movement [48,49]. The partial recovery in the tempering of IQT-DP steel in this work should be the result of suppression of lath boundary migration and hindering of dislocations movement by the fine inter-lath plate-like carbide at low-temperature tempering (Figure 8c). The fine plate-like carbides were replaced by coarser particles of cementite, which led to a rapid decrease in dislocation densities during high-temperature tempering (Figure 9c).

According to the above results, partial recovery and high-density dislocations were maintained even after tempering at 723 K. In addition, fine (quasi-) plate-like or sphere carbides were formed after tempering up to 723 K. This leads to an even larger YS than that of the IQ-DP case (Figure 4b).

**Figure 10:**
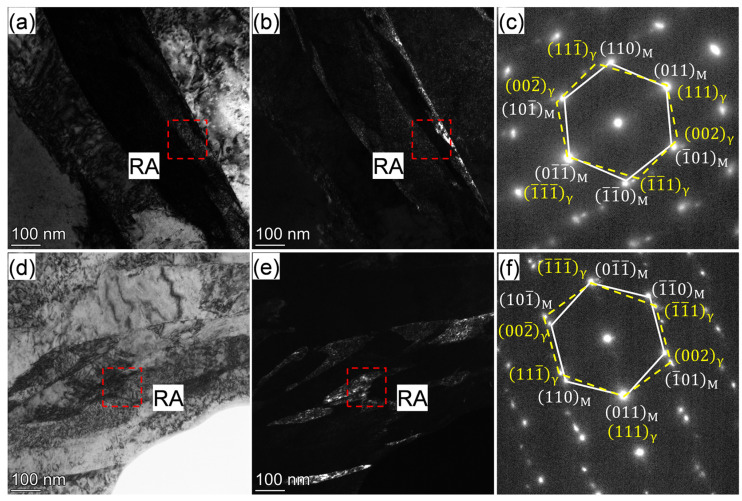
(**a**,**d**) BF, (**b**,**e**) DF TEM image and (**c**,**f**) selected area electron diffraction (SAED) patterns of (**a**–**c**) IQ-DP steel after intercritical annealing at 1033 K and (**d**–**f**) IQT-DP steel after tempering at 623 K.

Besides the ferrite matrix and martensite, a small amount of retained austenite (RA) was observed along the lath boundaries in the IQT-DP steel, as shown in the bright-field (Figure 10a,d) and dark-field (Figure 10b,e) images. The selected area electron diffraction (SAED) patterns and indexing are illustrated and displayed in Figure 10c and Figure 10f, respectively. Diffraction spots from [1–11]_bcc_ martensite zone axes and RA were clearly observed, holding a near Kurdjumov-Sachs (K-S) orientation relationship of (111)_fcc_∥(011)_bcc_, [1–10]_fcc_∥[1–11]_bcc_. Interlath RA is generally formed in the martensite of medium and high-carbon steels after quenching [50,51]. C and Mn are reported to enhance the formation of interlath RA [52]. Thus, the C and Mn enrichment of the acicular-shaped fresh martensite (Figure 7 and Table 2) enhanced the formation and stability of RA for the IQT-DP steel, and the RA was maintained even after tempering at 623 K. As for the alloy that shared a similar bulk chemical composition, the RA has been decomposed after tempering at the same temperature [45].

**Figure 11:**
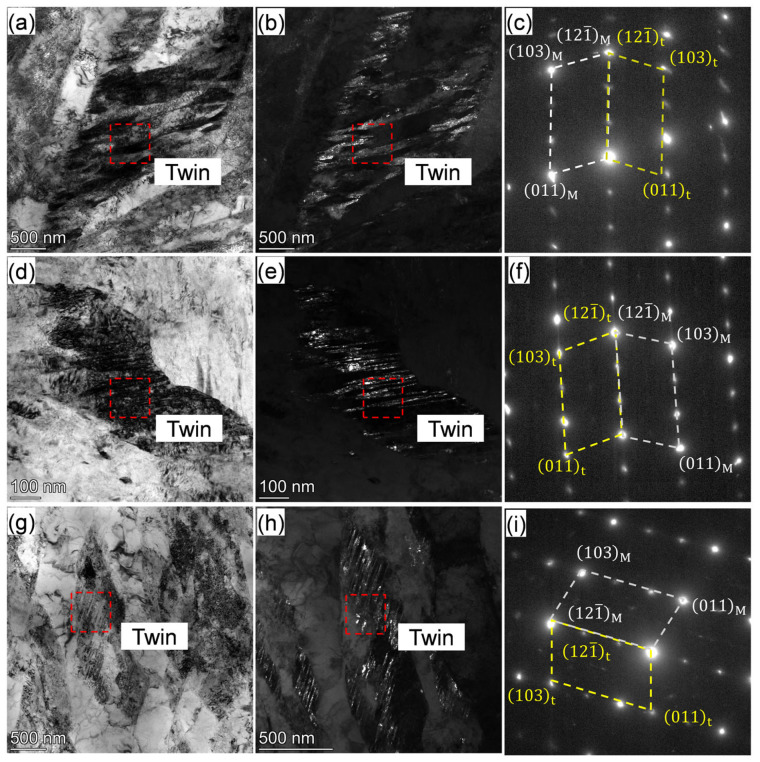
(**a**,**d**,**g**) BF, (**b**,**e**,**h**) DF TEM image and (**c**,**f**,**i**) SAED patterns of IQT-DP steels after intercritical annealing at 1033 K and tempering at varying temperatures of (**a**–**c**) 623 K, (**d**–**f**) 673 K, and (**g**–**i**) 723 K.

It was also noteworthy that the nanotwins were clearly observed in the IQT-DP steels after tempering at 623 K to 723 K as well, as shown by the bright/dark field images and diffraction spots of the twin structure in Figure 11a–i. This also contributes to the increase in strength [53], thus the resistance to tempering softening.

**Figure 12:**
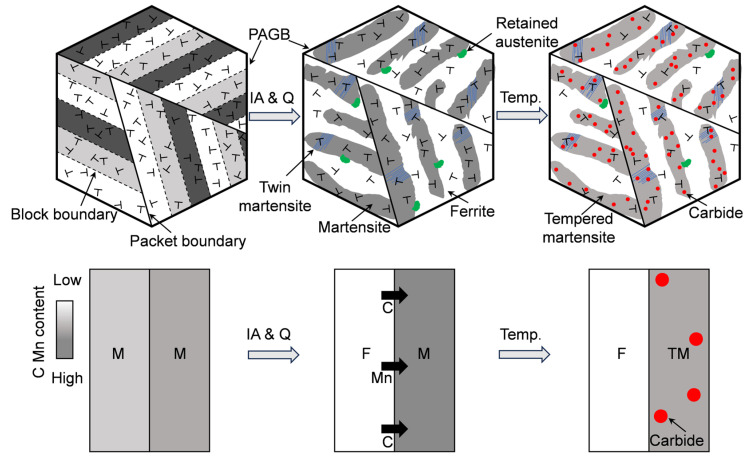
Schematic illustration of chemical enrichment, dislocation, and carbide precipitation evolution after intercritical annealing (IA) and tempering (Temp.) treatments.

The schematic illustrations of microstructure evolution for the IQ-DP steel just after intercritical annealing and subsequent tempering are shown in Figure 12. Alloying elements of Mn and C are enriched into the reverted austenite during the intercritical annealing, and the high-density dislocation and nano-twined acicular-shaped fresh martensite were formed after IA treatment and quenching. After tempering, the internal stress was released, plate-like or quasi-spherical fine carbides were precipitated, and the relatively high-density dislocations were maintained due to possible delay of lath structure recovery. In addition, nanotwins and very small amounts of RA existed due to the high Mn and C contents of the acicular-shaped martensite.

Hence, high-density dislocations, fine-carbide precipitates, and partially twinned tempered martensite were formed in the tempered martensite of the medium carbon Fe-C-Mn-Si lean alloy steel after intercritical quenching and tempering. These contributed to the resistance to tempering, softening, and strengthening of IQT-DP steel. Furthermore, the regular and uniform tempered martensite/ferrite structure ensured high ductility. On the other hand, the conventional low-alloy DP steels are characterized by the equiaxed martensite/ferrite structure, and local stress concentrations easily occur, resulting in low low-ductility [54,55].

## 5. Conclusions

In this work, the possibility of developing a low-cost Fe-C-Mn-Si lean-alloy fibrous dual-phase (DP) steel without adding expensive micro-alloying elements while using a medium-carbon approach was studied. The main findings are summarized as follows:

1. Fibrous DP steel was obtained via partial reversion from martensite. As the annealing temperature increased above 993 K, the strength gradually increased beyond 1300 MPa, while the ductility dramatically decreased and was below 6%, which is attributed to the increment in the amount of fresh-martensite formation.
2. The IQ-DP steel, after tempering, exhibited an increment in ductility with an acceptable loss in ultimate tensile strength of above 1200 MPa. The yield strength of the IQT-DP steels is even higher than that of the IQ-DP case as the tempering temperature is increased until 723 K. At the strength level of 1200 MPa–1300 MPa, the total elongation reached as high as 13%. The mechanical properties were beyond the current commercialized, and the literature reported low-alloy DP steels that used the low-cost Fe-C-Mn-Si lean alloy system without adding expensive alloying elements.
3. Plate-like or quasi-spherical fine carbides were precipitated, and the relatively high-density dislocations were maintained due to possible delay of lath recovery. In addition, nanotwins and a very small amount of RA existed due to the high Mn and C contents of the acicular shape martensite. These contribute to the resistance to tempering softening while keeping relatively high ductility. Quantitative strengthening models and calculations were not included in the present work, which is an interesting topic and will be studied in the future.

## Figures and Tables

**Table 1:** Chemical composition of the alloy [30] used in this study (mass%).

C	Mn	Si	S	P	Al	Fe
0.34	2.51	1.47	0.002	0.0048	0.0028	Bal.

**Table 2:** Element content (mass%) in martensite after intercritical annealing at 1033 K.

Element	Ortho	Experiment (Average)
C	0.40	-
Mn	2.76	3.19
Si	1.42	1.33

## Data Availability

The original contributions presented in this study are included in the article. Further inquiries can be directed to the corresponding author.
